# Dangerous Placebo During the COVID-19 Pandemic: A Series of Homoeopathic Arsenicum Album-Induced Liver Injury

**DOI:** 10.7759/cureus.26062

**Published:** 2022-06-18

**Authors:** Arif H Theruvath, Resmi Raveendran, Cyriac A Philips

**Affiliations:** 1 Clinical Research Division, Complementary and Alternative Medicine (Homoeopathy), The Liver Institute, Center of Excellence in Gastrointestinal Sciences, Rajagiri Hospital, Kochi, IND; 2 Clinical Research Division, Complementary and Alternative Medicine (Ayurveda), The Liver Institute, Center of Excellence in Gastrointestinal Sciences, Rajagiri Hospital, Kochi, IND; 3 Clinical and Translational Hepatology, Monarch Liver Laboratory, The Liver Institute, Center of Excellence in Gastrointestinal Sciences, Rajagiri Hospital, Aluva, IND

**Keywords:** drug-induced liver injury (dili), complementary and alternative medical (cam), acute-on-chronic liver failure (aclf), non-alcoholic steatohepatitis (nash), immune booster, arsenic, acute liver injury, cirrhosis, portal hypertension

## Abstract

Complementary and alternative medicines were promoted as health supplements, “immune-boosters” and COVID-19 preventive drugs through visual, print, and social media, during the pandemic. In this context, specifically in India, the homeopathic remedy, Arsenicum Album 30C prepared from arsenic trioxide was widely prescribed and publicly supplied through government agencies among adults and school-going children. Inorganic arsenic, known as the “king of poisons” is a highly toxic substance with the potential to cause acute as well as chronic injury to multiple organ systems, mainly skin, lung, liver, and kidneys. Acute liver injury due to arsenic-containing formulations is seldom reported. We present three cases of acute liver injury, leading to death in one patient with underlying non-alcoholic steatohepatitis (NASH) cirrhosis, after consumption of the homeopathic remedy AA30 for COVID-19 prevention.

## Introduction

The novel coronavirus disease 2019 (COVID-19) challenged the scientific community to discover preventive and therapeutic measures to ameliorate morbidity and mortality associated with the unanticipated pandemic. In developing countries, complementary and alternative medical (CAM) systems were promoted and pushed to the forefront via visual, print, and social media, as “supposed” COVID-19 preventive measures that were advertised as immune-boosters (IB). In India, a homeopathic remedy, Arsenicum Album 30C (AA30, from arsenic trioxide) was widely promoted, prescribed, and publicly supplied through government agencies from house to house as an IB and COVID-19 preventive in adults and school-going children [1]. The Indian Ayush Ministry guideline recommended dose of AA30 for COVID-19 “prevention” is four pills or three drops of liquid formulation in one spoon of water for three consecutive days, repeated every 21 days until “end of pandemic” [2]. We report a novel association of acute severe liver injury attributable to AA30 in three patients.

## Case presentation

Case one

A 70-year-old man with compensated non-alcoholic steatohepatitis (NASH)-related cirrhosis and diabetes mellitus consumed the homeopathic IB AA30 as prescribed for 12 weeks prior to the onset of symptoms. He presented with jaundice and abdominal distension within four weeks after the onset of loss of appetite and well-being. The patient was not on any other hepatotoxic agents, over-the-counter medications, or herbal and dietary supplements. Investigations revealed the presence of conjugated hyperbilirubinemia, ascites, and abnormal coagulation, suggestive of acute-on-chronic liver failure (ACLF). Further investigations to identify known causes of acute deterioration of underlying cirrhosis were performed, including a transjugular liver biopsy. All competing causes for acute liver injury were meticulously ruled out. These included infections-tests for immunoglobulin M (IgM) for viral hepatitis A and E; hepatitis B surface antigen and IgM antibody to hepatitis B core antigen; nucleic acid tests via polymerase chain reaction for hepatitis C; IgM for herpes zoster and herpes simplex, cytomegalovirus, parvovirus, Epstein-Barr virus. Complete auto-antibody testing for autoimmune hepatitis (AIH) was negative. The Roussel Uclaf Causality Assessment (RUCAM) demonstrated “probable” (score 7) drug-induced liver injury (DILI) and simplified AIH score was less than 5, revealing the cause of acute liver injury leading to ACLF as the homeopathic remedy, AA30. The liver biopsy revealed multiacinar hepatocyte necrosis, lymphocytic, neutrophilic, and eosinophilic inflammation in the absence of interface hepatitis, which were predominantly portal-based in the background of cirrhosis, suggestive of DILI. Analysis of drugs consumed could not be performed in view of inadequate sample availability. The patient and family consented to arsenic analysis in nail and hair samples which revealed extremely high levels of the heavy metal, supportive of arsenic toxicity and associated liver injury in the patient. Evaluation of hair and hair samples of two family members (below detection limits, method detection limit being 0.1 mg/kg), staying in the same household did not reveal levels signifying cluster arsenic poisoning from water or soil sources. The patient succumbed to complications related to ACLF, nine months after the initial diagnosis.

Case two

A 68-year-old male with systemic hypertension controlled on telmisartan who ingested AA30 as prescribed for four weeks prior to the onset of symptoms. There was no associated jaundice or cholestatic symptoms, but liver tests revealed acute hepatitis with an elevation of liver enzymes. The patient was not on any other hepatotoxic agents, over-the-counter medications, or herbal and dietary supplements. Further investigations did not reveal the presence of underlying chronic liver disease or portal hypertension. All competing causes for acute liver injury were meticulously ruled out similar to the extensive workup that was done in case one. The RUCAM demonstrated “probable” (score 8) DILI and simplified AIH score was less than 5, revealing the cause of acute non-icteric hepatitis as the homeopathic remedy, AA30. The liver biopsy revealed perivenular hepatocyte necrosis, with predominantly portal-based mixed cellular inflammation consisting of plasma cells, eosinophils, lymphocytes, and scattered neutrophils. Additionally, ballooning of hepatocytes was marked with scattered rosettes and moderate interphase hepatitis in the presence of mild portal and sinusoidal fibrosis suggestive of DILI. Acute hepatitis resolved after drug withdrawal and finite course of steroids within three months, without any recurrence on follow-up.

Case three

**Figure 1:**
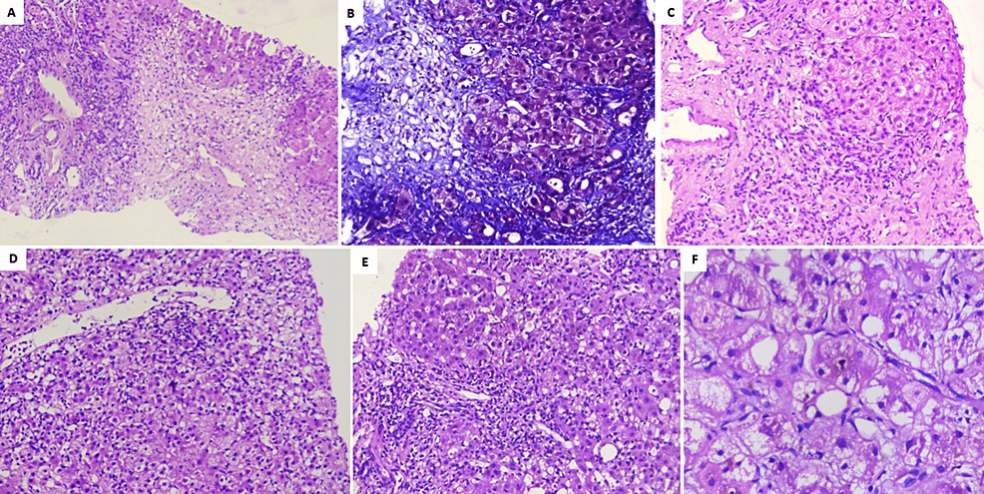
Liver histopathology of patients with Homeopathic remedy Arsenicum Album 30C-related liver injury A) Extensive zone-3 hepatocyte necrosis in patient A (hematoxylin and eosin, 40×); B) deep staining of perisinusoidal fibrosis and pale blue staining of necrotic regions notable in patient B (Masson-trichrome, 100×); C) hepatocyte nodule with ballooned hepatocytes with ductular reaction, mixed lymphocytic, neutrophilic inflammation in patient A (hematoxylin and eosin, 40×); D) perivenular hepatocyte necrosis along with lymphocytic and neutrophilic inflammation in patient C (hematoxylin and eosin, 100×); E) severe portal inflammation with neutrophilic and lymphocytic infiltrates in patient B (hematoxylin and eosin, 100×); F) ballooned hepatocytes with canalicular cholestasis in patient C (hematoxylin and eosin, 200×)

A 48-year-old overweight woman consumed homeopathic AA30 pills as COVID-19 preventive for one week prior to the onset of her symptoms of cholestatic jaundice. Prior to the development of jaundice, she had nonspecific gastrointestinal symptoms such as nausea and progressive loss of appetite. Liver tests revealed conjugated hyperbilirubinemia with highly raised liver enzymes. The patient was not on any other hepatotoxic prescription drugs, over-the-counter medications, or herbal and dietary supplements. Further investigations did not reveal the presence of underlying chronic liver disease or portal hypertension. All competing causes for acute liver injury were meticulously ruled out similar to the extensive workup that was done in case one. The RUCAM demonstrated “probable” (score 7) DILI and simplified AIH score was less than 5, revealing the cause of acute cholestatic hepatitis as the homeopathic remedy, AA30. The liver biopsy revealed spotty, focal hepatocyte necrosis, with predominantly portal-based neutrophilic and eosinophil-rich inflammation, moderate steatosis, and mild interface hepatitis with underlying mild perisinusoidal fibrosis, suggestive of DILI. The acute cholestatic hepatitis resolved after drug withdrawal and a finite course of steroids within six months, without any recurrence on follow-up. Chemical analysis and toxicology (inductively coupled optical emission spectroscopy and triple-quadrupole gas chromatography with tandem mass spectroscopy method) on two sets of AA30 samples retrieved from case three revealed D-mannose, and melezitose, and arsenic respectively, demonstrating batch-to-batch variation due to poor manufacturing practices. The pertinent, representational liver biopsy findings are shown in Figure 1.

The at-presentation clinical, investigational parameters, pertinent treatments, and clinical outcomes of all three patients are shown in Table 1.

**Table 1:** The clinical, investigational, treatment, and clinical parameters of all three patients NASH: non-alcoholic steatohepatitis; ANA: antinuclear antibody; ASMA: anti-smooth muscle antibody; LKM: liver-kidney-microsomal antibody; LC: antibody to liver cytosol; IgG: immunoglobulin G; AST: aspartate aminotransferase; ALT: alanine aminotransferase; ALP: alkaline phosphatase; INR: international normalized ratio; RUCAM: Roussel Uclaf Causality Assessment Method; AIH: autoimmune hepatitis

Variable/feature	Case one	Case two	Case three
Age	70 years	68 years	48 years
Gender	Male	Male	Female
Diabetes	Yes (controlled)	No	No
Systemic hypertension	No	Yes	No
Hypothyroidism	No	No	No
Cardiac disease	No	No	No
Underlying liver disease, etiology	Yes, NASH	No	No
Concomitant medications	Metformin, glimepiride	Telmisartan	None
Duration of use	6 years	8 years	-
ANA, ASMA, anti-LKM1, anti-LC1	Negative	Negative	Negative
Serum total IgG (normal 600–1600 mg/dl)	1456	1924	1398
Homeopathy remedy formulation	Globules and liquid	Globules	Globules
Duration of use (days)	86	32	10
Time to onset of symptoms from starting drug (days)	92	42	15
Time to normalization of bilirubin	>50% reduced in 21 days, not normalized	-	8 days
Time to normalization of liver enzymes	21 days	8 days	12 days
Jaundice	Yes	No	Yes
Ascites	Yes	No	No
Cholestasis	No	No	Yes
Hemoglobin(13.2 to 16.6 g/dL)	9.5 g/dL	14.2 g/dL	11.4 g/dL
Total leucocyte count (4,000 to 11,000 cells/mm^3^)	12600 cells/mm^3^	5200 cells/mm^3^	8800 cells/mm^3^
Platelet count (150,000 to 400,000 cells/mm^3^)	83000 cells/mm^3^	226000 cells/mm^3^	254000 cells/mm^3^
Total bilirubin (0.8 to 1.2 mg/dL)	15 mg/dL	2.2 mg/dL	6.8 mg/dL
AST (10 to 40 U/L)	148 U/L	498 U/L	898 U/L
ALT (7 to 56 U/L)	161 U/L	473 U/L	1122 U/L
ALP (44 to 147 U/L)	212 U/L	188 U/L	364 U/L
Serum creatinine (0.7 to 1.3 mg/dL)	0.4 mg/dL	0.9 mg/dL	1.1 mg/dL
Serum sodium (135 to 145 mEq/L)	130 mEq/L	138 mEq/L	136 mEq/L
INR (below 1.1)	2.92	0.96	1.2
RUCAM score	7 (probable)	8 (probable)	7 (probable)
Revised original score for AIH	< 5 (possible)	< 5 (possible)	< 5 (possible)
Treatment details	Best supportive care, no steroids	Prednisolone + azathioprine, tapered over 6 months	Prednisolone tapered over 3 months
Final outcome	Died (9 months)	Alive	Alive

The liver histology findings of all three patients are shown in Table 2.

**Table 2:** Liver biopsy findings of all three patients who developed drug-induced liver injury after consuming the homeopathic remedy, Arsenicum Album 30C

Liver biopsy findings	Case one	Case two	Case three
Neutrophils	++	+	++
Lymphocytes	+++	++	+
Eosinophils	++	++	++
Plasma cells	No	+++	No
Pattern of injury	Hepatocellular	Hepatocellular	Hepatocellular/cholestatic
Necrosis	Multiacinar	Bridging, perivenular	Spotty, focal
Steatosis	Moderate	Mild	Moderate
Ballooning	Moderate	Severe	Severe
Rosetting	No	Scattered	No
Interface hepatitis	No	Moderate	Mild
Cholestasis	No	No	Moderate
Type of inflammation	Portal-based	Portal-based	Portal-based, lobular+
Fibrosis type	F4, cirrhosis	Mild sinusoidal/portal	Mild perisinusoidal

Table 3 shows chemical and toxicology analyses performed on the three patients.

**Table 3:** Pertinent chemical and toxicology analyses results in the patients *Heavy metal concentration was determined by an inductively coupled plasma-atomic emission spectrometer (IRIS Intrepid II XSP Duo; Thermo Electron Corp., Munich, Germany) using chemical standards, reagents, and vials per the United States Environmental Protection Agency standards, methods 5021A, 8015, 8021, and 8260. Full-scan qualitative analysis for inorganic and organic compounds was performed using triple-quadrupole gas chromatography coupled with tandem mass spectrometry (GC-MS/MS; Thermo Fisher Scientific, Waltham, MA, USA).

Chemical analysis and toxicology*	Case one	Case two	Case three
Retrieved homeopathic drug	No, fully consumed	No, fully consumed	Yes (2 samples)
Heavy metal	-	-	Arsenic 0.18 mg/kg
Other compounds identified	-	-	Melezitose, D-mannose
Arsenic in hair sample (values >1 mg/kg indicates excessive exposure)	7.54 mg/kg	-	-
Arsenic in nail sample (fingers; normal range is reported to be 20-200 µg/kg)	13.26 mg/kg	-	-
Arsenic in nail sample (toes; normal range is reported to be 20-200 µg/kg)	5.15 mg/kg	-	-

Cases one and two patients were vaccinated against COVID-19 infection with a single dose while case three did not undergo vaccination at all prior to symptomatic presentation. The first patient did not complete the vaccination schedule, while case two completed the second dose of vaccine, and case three initiated scheduled vaccination on follow-up of 2-3 weeks after complete resolution of DILI.

## Discussion

Complementary and alternative medicines, specifically Ayurvedic and Homeopathic supplements were promoted as COVID-19 preventive drugs during the pandemic. The homeopathic remedy, AA30 prepared from mother compound arsenic trioxide was touted as an “immunity booster” and “vaccine-equivalent” by alternative medicine practitioners, was supplied via government agencies among adults and school-going children as a preventive measure. Acute liver injury due to arsenic-containing formulations is reported rarely. We presented three cases of acute hepatitis leading to death in one patient with underlying NASH cirrhosis, after consumption of the homeopathic remedy AA30 for COVID-19 prevention. We have meticulously excluded all other major competing causes for acute liver injury in our cohort of patients, including underlying AIH through systematic clinical, investigational, and histopathology evaluations. Homeopathic medicines in high dilutions, even though ineffective for any disease condition, are probably safe and unlikely to provoke severe adverse reactions. Nonetheless, systematic reviews have shown that Homeopathy has the potential to harm patients and consumers in both direct and indirect ways. The incidence of adverse effects of homeopathic drugs was not uncommon and was at times greater than placebo in some controlled clinical trials [3,4]. Arsenic toxicity from Homeopathic drugs is well described in the literature, but the acute liver injury is seldom reported [5-7]. Arsenic occurs in two oxidative forms-the trivalent arsenite and arsenate, the pentavalent form. The former is 60 times more toxic than the latter. Organic arsenic is nontoxic whereas inorganic arsenic (arsenic trioxide, the mother compound used in homeopathic AA30) is toxic. Arsenic toxicity occurs in the presence of reactive oxygen intermediates generation during redox cycling and metabolic activation processes resulting in lipid peroxidation. Arsenic trioxide binds thiol or sulfhydryl groups in tissue proteins of the liver, lungs, kidney, gastrointestinal mucosa, and keratin-rich tissues such as skin, hair, and nails [6,7]. Acute severe exposure to large amounts of arsenical compounds produces predominantly gastrointestinal symptoms which was classically absent in our patients except for case three who presented with liver and gastrointestinal symptoms within a short duration of exposure. Exposure to inorganic arsenic through drinking water is a major public health problem in both developing and developed countries. The United States Environmental Protection Agency’s safe cut-off value for arsenic exposure in drinking water is 10 μg/L. Drugs and health supplements are ideally not supposed to contain arsenic and hence safe limits or cut-off remain undefined. In acute exposure, the maximal deposition of arsenic occurs within the kidneys and liver and in the hair and nails after two weeks of ingestion. Arsenic toxicity leads to hepatomegaly, steatosis, hepatocyte necrosis, and portal fibrosis and arsenic exposure in steatotic livers is associated with necro-inflammatory changes and progressive liver damage, a notable finding in our group of patients with underlying fatty liver disease [8]. The liver is usually vulnerable to prolonged exposure to small amounts of arsenic. Nonetheless, liver-related injury and response to arsenic from person to person cannot be predicted due to idiosyncratic type of injury. The earliest description of arsenic-induced liver disease was reported by Bang in the 18th century in a patient who developed ascites due to prolonged use of therapeutic doses of liquid arsenic. Subsequently, an outbreak of liver disease among beer drinkers in the north of England, where arsenopyrite had been used to produce beer from starch was described by a group of investigators. Acute liver injury and jaundice after consuming therapeutic doses of arsenicals, similar to the current patient cohort, were also described in 1945 [9]. AA30 was found ineffective as a preventive of COVID-19 infection in a randomized placebo-controlled trial [10].

In our series, certain limitations require further deliberation. AA30 as a definite cause of DILI could not be ascertained due to lack of rechallenge which is also currently not recommended by experts. The empirical decision to not use steroids in the first case was due to the presence of underlying cirrhosis, suspected secondary bacterial infection, and diabetes mellitus in the elderly gentleman. A diagnosis of DILI does not require a demonstration of the potential toxic agent in the affected patient and a causality assessment, even in the presence of its limitations, is a strong and accepted method to identify drug-related liver damage along with the exclusion of competent causes. Even though other members of the family in case one consumed AA30, this was only for a very short duration, unlike the patient, who consumed it for close to 90 days.

## Conclusions

Health regulatory authorities, physicians, general and patient population must be aware of the potential harms associated with the large-scale promotion of untested, alternative medical systems during a medical emergency so as to prevent an “epidemic” of avoidable DILI within the ongoing pandemic. Even though ultra-diluted homeopathic remedies, found ineffective as shown in large-scale meta-analysis, are considered safe for use due to the absence of any active compound beyond 12C dilution. Nonetheless, poor manufacturing practices, use of concentrated tincture formulations, and adulteration and contamination of homeopathic remedies can still pose considerable toxicity in predisposed persons. From a scientific and evidence-based standpoint, it is imperative that the general population and at-risk persons understand that vaccination, and not untested, misleading IBs, remains the best available armamentarium against COVID-19 which helps in fighting back the pandemic.
